# Is Ankyrin a genetic risk factor for psychiatric phenotypes?

**DOI:** 10.1186/1471-244X-11-103

**Published:** 2011-06-24

**Authors:** Alejandro Gella, Mònica Segura, Núria Durany, Bruno Pfuhlmann, Gerald Stöber, Micha Gawlik

**Affiliations:** 1 Faculty of Medicine and Health Sciences, Universitat Internacional de Catalunya, Josep Trueta s/n, Sant Cugat del Vallès-08195, Spain; 2 Department of Psychiatry, Psychosomatics and Psychotherapy, University of Würzburg, Füchsleinstraße 15, Würzburg-97080, Germany

## Abstract

**Background:**

Genome wide association studies reported two single nucleotide polymorphisms in *ANK3 *(rs9804190 and rs10994336) as independent genetic risk factors for bipolar disorder. Another SNP in *ANK3 *(rs10761482) was associated with schizophrenia in a large European sample. Within the debate on common susceptibility genes for schizophrenia and bipolar disorder, we tried to investigate common findings by analyzing association of *ANK3 *with schizophrenia, bipolar disorder and unipolar depression.

**Methods:**

We genotyped three single nucleotide polymorphisms (SNPs) in *ANK3 *(rs9804190, rs10994336, and rs10761482) in a case-control sample of German descent including 920 patients with schizophrenia, 400 with bipolar affective disorder, 220 patients with unipolar depression according to ICD 10 and 480 healthy controls. Sample was further differentiated according to Leonhard's classification featuring disease entities with specific combination of bipolar and psychotic syndromes.

**Results:**

We found no association of rs9804190 and rs10994336 with bipolar disorder, unipolar depression or schizophrenia. In contrast to previous findings rs10761482 was associated with bipolar disorder (p = 0.015) but not with schizophrenia or unipolar depression. We observed no association with disease entities according to Leonhard's classification.

**Conclusion:**

Our results support a specific genetic contribution of *ANK3 *to bipolar disorder though we failed to replicate findings for schizophrenia. We cannot confirm *ANK3 *as a common risk factor for different diseases.

## Background

Schizophrenia and bipolar disorder are genetically complex diseases with numerous proposed genetic risk factors encompassing different pathophysiological pathways of neurotransmission, brain development or synaptic plasticity with each small contribution to disease risk and inconsistent results among replication studies (Stöber et. al 2009) [1,2]. Recently genome wide association studies (GWAs) lead to identification of new susceptibility genes with genome-wide levels of significance: zinc finger gene *ZNF804A *on chromosome 2q32 or the *MHC*-locus at 6p21 on schizophrenia. For bipolar disorder the most promising results have been reported for *CACNA1C *and *ANK3 *(ankyrin 3, node of Ranvier) [3-5]. Subsequently *CACNA1C *and *ZNF804A *were proposed as common risk variants for both bipolar disorder and schizophrenia and a Meta-analysis additionally added the *MHC*-locus as a common risk factor for both diseases [5].

*ANK3 *at 10q21.2 consists of 44 exons spanning ~700 kb on genomic DNA with multiple splicing variants. A GWA study based on pooled DNA found association with bipolar disorder and rs9804190 located intronic between exon 36 and 37 at the locus *ANK3 *[6]. A Meta-analysis of GWA on bipolar patients with European ancestry reported an additional marker rs10994336, about 340 kb distal to rs9804190, at the 3-UTR of *ANK3 *[7]. Further analysis suggested that each variant might contribute independently to bipolar disorder [8]. A further SNP located 3-UTR showed suggestive evidence of genome-wide association in a Han Chinese sample [9]. Subsequent studies found a genetic marker at *ANK3 *to be associated with schizophrenia as well. Analysis in a GWA study of a Norwegian discovery sample with a large European replication sample reported association of rs10761482 located near 3-UTR between exon 41 and 42 with disease at a distance of 84.5 kb to rs9804190 [10].

Ankyrin 3 is a brain expressed member of a protein-family linking the integral membrane proteins to the underlying spectrin-actin cytoskeleton. The gene product Ankyrin-G of 4377 amino acids locates on axonal initial segment and at nodes of Ranvier in the central and peripheral neurons. Ankyrin-G is proposed to play a regulatory role on sodium channel function, cell adhesion and neuronal development [11-16]. A post mortem study reported reduced immunoreactive of Ankyrin-G in pyramidal neurons in the superficial cortical layer of the dorsolateral prefrontal cortex in subjects with schizophrenia [17].

Within the debate on common susceptibility genes for schizophrenia and bipolar disorder we attempted to replicate common findings of a genetic association for different disease entities by analyzing association of *ANK3 *with major psychosis in a case-control study with SNPs rs9804190, rs10994336, and rs10761482. For diagnosis we used beside ICD10 Leonhard's classification separating disease entities with specific combination of bipolar and psychotic syndromes [18].

Karl Leonhard divides psychoses into five main groups, systematic schizophrenias, unsystematic schizophrenias and cycloid psychoses. Affective psychoses are subdivided into bipolar manic depression and monopolar depression. In family and twin studies based on Leonhard's classification, a different genetic background for each diagnostic category was demonstrated [19]

## Methods

Index cases were recruited from the Department of Psychiatry, Psychosomatics and Psychotherapy at of the University of Würzburg. The sample encompassed 920 cases (631 males, 68%) with psychosis according to ICD10 for schizophrenia or related diseases with an average age at onset of 26.5 years and an average age at recruitment of 41 years including 182 cases with schizoaffective disorder (ICD10 F20-F25). 400 cases (231 males, 58%) with bipolar disorder (F30-F31) with an average age at onset of 32 years and an average age at recruitment of 42.5 years and 220 cases (134 males, 61%) with unipolar depression (F32-F33). with an average age at onset of 43 years and an average age at recruitment of 51 years.

Sample was further subdivided according to Leonhard's classification systematic schizophrenias (n = 228), unsystematic schizophrenias (n = 635), cycloid psychosis (n = 309), manic depression (n = 284) and monopolar depression (n = 90) [17]. Diagnosis in differentiated psychopathology was made by repeated personal examinations of experienced psychiatrists (BP, MG, GS).

The 480 volunteer control subjects (283 males, 59%) were recruited from the blood donor centre at the University of Würzburg. The average age of recruitment was 29 years. The preponderance of males in both samples avoided gender distortion in comparison of cases and controls. All subjects were unrelated and of German Caucasian descent. The Ethics Committee of the University of Würzburg had approved the study, and written informed consent was obtained from all subjects.

PCR for allelic discrimination was performed in a final reaction volume of 20 μl containing 20 ng genomic DNA and 10 μl of 2 × TaqMan^®^Universal PCR Master Mix (Applied Biosystems) and 1 μl of 20 × TaqMan™ SNP genotyping assay including fluorescent tags specific for the wild type allele and the variant allele. Marker amplification was performed in microtiter plates on Biometra thermocyclers (Whatman). PCR amplification conditions were according to the manufacturer's recommendation [10 min at 95°C followed by 15 sec at 92°C and 60 sec at 60°C for 40 cycles]. Allelic discrimination with endpoint detection of fluorescence was performed at 60°C on an ABI prism 7000 sequence detection system followed by analysis with an appropriate software package (Applied Biosystems). All genotype experiments were made at least in duplicate, with quality control of automated allele calling by two independent operators blind to phenotype. The calling rate was 99%.

Software FAMHAP was used to test for association [20]. Hardy-Weinberg equilibrium (HWE) and pairwise standardized linkage disequilibrium (LD) were calculated with the program HAPLOVIEW [21]. The software "stastical power calculator" was used analyzing power for association test [22].

## Results

Corresponding to HAPMAP data rs9804190 locates between LD-block 7 and 8, rs10761482 in LD-block 26 and rs10994336 in a downstream LD block of *ANK3*. Thus, linkage disequilibrium (LD) was low between the analyzed markers with LD' 0.018 between rs9804190 and rs10761482, 0.0060 between rs9804190 and rs10994336 and 0.72 between rs10761482 and rs10994336 located at 3'-UTR. All SNPs were in HWE.

Analyzing Allele and genotype frequencies in cases according to the ICD 10 classification with schizophrenia, bipolar disorder or major depression revealed no association for SNPs rs9804190 and rs10994336, (table 1 and 2). We observed no significant difference between cases and controls for subgroup with schizoaffective disorder. SNP rs10761482 was associated with bipolar disorder (p = 0.015, OR 1.304, CI 1.065 - 1.595) but not with schizophrenia, nor with subgroup schizoaffective disorder nor with unipolar depression (table 1 and 2).

**Table 1:** Bipolar disorder according to ICD10: Genotype distribution and test for association

	Cases	Controls	Cases	Controls		Cases	Controls		Cases	Controls	
SNP	n	n	CC	CC	P	CT	CT	P	TT	TT	P
**rs9804190 (C/T)**	400	480	0.618	0.578	0.237	0.327	0.380	0.101	0.056	0.042	0.337
**rs10994336 (C/T)**	400	480	0.843	0.874	0.182	0.154	0.119	0.13	0.003	0.006	0.415
**rs10761482 (C/T)**	400	480	0.652	0.572	0.015	0.300	0.359	0.063	0.048	0.069	0.19

P: test for association (FAMHAP); CC, CT, TT: genotypes

**Table 2:** Schizophrenia according to ICD10: Genotype distribution and test for association

	Cases	Controls	Cases	Controls		Cases	Controls		Cases	Controls	
SNP	n	n	CC	CC	P	CT	CT	P	TT	TT	P
**rs9804190 (C/T)**	920	480	0.577	0.578	0.949	0.369	0.380	0.674	0.055	0.042	0.284
**rs10994336 (C/T)**	920	480	0.884	0.874	0.617	0.112	0.119	0.689	0.004	0.006	0.62
**rs10761482 (C/T)**	920	480	0.557	0.572	0.593	0.394	0.359	0.196	0.048	0.069	0.113

P: test for association (FAMHAP); CC, CT, TT: genotypes

Sample was further differentiated according to Leonhard's classification. Analyzing association of SNPs with schizophrenic spectrum divided into subgroups systematic schizophrenias, unsystematic schizophrenias and cycloid psychosis provided no significance. Likewise affective diseases with manic depression and monopolar depression reached no significant association.

Analyzing haplotype with FAMHAP provided no further risks haplotype concordant with observed low LD.

Our study population with 1540 cases and 480 controls had a power of 55.1% to replicate the reported association with bipolar disorder and of 69.1% with schizophrenia (alpha = 0.05%).

## Discussion

Common susceptibility genes for schizophrenia and bipolar disorder challenge traditional diagnostic categories and boundaries between schizophrenia and bipolar disorder. We attempt to replicate genetic association findings of *ANK3 *as a possible common risk factor for schizophrenia and affective disorders in a case control study of > 2000 subjects of German descent. Analysis of previous associated SNPs in different LD-Blocks, located intronic (rs9804190 and rs10761482) or 30 kb downstream of *ANK3 *(rs10994336) found a nominally significant association of SNP rs10761482 with bipolar disorder (p = 0.015, OR 1.304) but not with schizophrenia (table 1 and 2). Thus, association of this marker with schizophrenia in a GWA analysis of European samples could not be confirmed [16].

We failed to confirm an association of rs9804190 and rs10994336 with bipolar disorder reported in two previous GWA studies. We found no association with unipolar depression or schizophrenia including subgroup of schizoaffective disorder (table 1 and 2) [13,14]. Analyzing haplotype provided no further risks haplotype concordant with observed low LD between the markers.

Our failure to replicate previous findings could be due to insufficient sample size. The study had a power of 55.1% to replicate reported association with bipolar disorder and of 69.1% with schizophrenia (alpha = 0.05%) [14,16]. However, in our study were cases and controls of the same genetic background, minimizing a distortion regarding genetic heterogeneity. The strength of our strategy is the combination of operational diagnostic criteria (ICD-10) and Leonhard's categorical diagnostic approach. In search for common risk factors for schizophrenia and bipolar disorder we found no association in the schizophrenic spectrum neither with systematic schizophrenias nor with subgroups with a specific combination of bipolar and psychotic syndromes: Particularly the unsystematic schizophrenias and strictly defined manic depression with strong genetic background [19,23,24]. Other disease entities according to Leonhard's classification were not associated to any of the markers.

Our data support findings from two meta-analyses of GWA-studies searching for common risk variants in *ANK3 *for schizophrenia, bipolar disorder or unipolar depression: One study combining Meta-analysis and additional genotyping of a bipolar and unipolar sample from the US, the UK, Ireland, and Netherlands, found no association of variants in *ANK3 *and unipolar depression. Another Meta-analysis on GWA studies based on schizophrenia and bipolar disorder cohorts with samples from UK observed no significant results for schizophrenia. Both Meta-analyses suggested a specific effect of *ANK3 *for bipolar disorder [25,5].

Since genetically associated SNPs around *ANK3 *are intronic or in downstream regions located, causative coding variants or associated haplotype blocks are still missing. Regard distorted gene regulation as patho-physiological causative factor a recent study reported evidence for cis-acting regulation of *ANK3 *by testing for allelic expression imbalance, but the study failed to attribute dysregulation to risk-associated SNPs [26].

## Conclusions

In conclusion, our results support a genetic contribution of *ANK3 *to ICD 10 bipolar disorder, though we failed to replicate findings for schizophrenia according to ICD 10 or Leonhard's classification. Our study cannot confirm *ANK3 *as a common risk factor for both diseases, challenging the hypothesis that bipolar disorder and schizophrenia are just different phenotypes of the same disease.

## Competing interests

The authors declare that they have no competing interests.

## Authors' contributions

AG and MS performed the experiments and drafted the manuscript, ND and GS conceived the study and participated in the coordination. BP and GS carried out the diagnostic evaluation of the patients; MG carried out the statistical analyses, coordinated the study and wrote the manuscript. All authors read and approved the final manuscript.

## Authors' information

To our deep regret Professor Núria Durany passed away on August 27th 2010 after an intense but short fighting an illness. We shall continue to cherish her passion for science.

## Pre-publication history

The pre-publication history for this paper can be accessed here:

http://www.biomedcentral.com/1471-244X/11/103/prepub

## References

[B1] Allen NC Bagade S McQueen MB Ioannidis JP Kavvoura FK Khoury MJ Tanzi RE Bertram L Systematic meta-analyses and field synopsis of genetic association studies in schizophrenia: the SzGene database Nat Genet 2008 40 827 834 10.1038/ng.171 18583979

[B2] Stöber G Ben-Shachar D Cardon M Falkai P Fonteh AN Gawlik M Glenthoj BY Grunblatt E Jablensky A Kim YK Kornhuber J McNeil TF Muller N Oranje B Saito T Saoud M Schmitt A Schwartz M Thome J Uzbekov M Durany N Riederer P Schizophrenia: From the brain to peripheral markers. A consensus paper of the WFSBP task force on biological markers World Journal of Biological Psychiatry 2009 10 127 155 10.1080/15622970902898980 19396704

[B3] Stefansson H Ophoff RA Steinberg S Andreassen OA Cichon S Rujescu D Werge T Pietiläinen OP Mors O Mortensen PB Sigurdsson E Gustafsson O Nyegaard M Tuulio-Henriksson A Ingason A Hansen T Suvisaari J Lonnqvist J Paunio T Børglum AD Hartmann A Fink-Jensen A Nordentoft M Hougaard D Norgaard-Pedersen B Böttcher Y Olesen J Breuer R Möller HJ Giegling I Rasmussen HB Timm S Mattheisen M Bitter I Réthelyi JM Magnusdottir BB Sigmundsson T Olason P Masson G Gulcher JR Haraldsson M Fossdal R Thorgeirsson TE Thorsteinsdottir U Ruggeri M Tosato S Franke B Strengman E Kiemeney LA Genetic Risk and Outcome in Psychosis (GROUP) Melle I Djurovic S Abramova L Kaleda V Sanjuan J de Frutos R Bramon E Vassos E Fraser G Ettinger U Picchioni M Walker N Toulopoulou T Need AC Ge D Yoon JL Shianna KV Freimer NB Cantor RM Murray R Kong A Golimbet V Carracedo A Arango C Costas J Jönsson EG Terenius L Agartz I Petursson H Nöthen MM Rietschel M Matthews PM Muglia P Peltonen L St Clair D Goldstein DB Stefansson K Collier DA Common variants conferring risk of schizophrenia Nature 2009 460 744 7 19571808 10.1038/nature08186 PMC3077530

[B4] O'Donovan MC Craddock N Norton N Williams H Peirce T Moskvina V Nikolov I Hamshere M Carroll L Georgieva L Dwyer S Holmans P Marchini JL Spencer CC Howie B Leung HT Hartmann AM Möller HJ Morris DW Shi Y Feng G Hoffmann P Propping P Vasilescu C Maier W Rietschel M Zammit S Schumacher J Quinn EM Schulze TG Williams NM Giegling I Iwata N Ikeda M Darvasi A Shifman S He L Duan J Sanders AR Levinson DF Gejman PV Cichon S Nöthen MM Gill M Corvin A Rujescu D Kirov G Owen MJ Buccola NG Mowry BJ Freedman R Amin F Black DW Silverman JM Byerley WF Cloninger CR Molecular Genetics of Schizophrenia Collaboration Identification of loci associated with schizophrenia by genome-wide association and follow-up Nat Genet 2008 40 1053 5 10.1038/ng.201 18677311

[B5] Williams HJ Craddock N Russo G Hamshere ML Moskvina V Dwyer S Smith RL Green E Grozeva D Holmans P Owen MJ O'Donovan MC Most genome-wide significant susceptibility loci for schizophrenia and bipolar disorder reported to date cross-traditional diagnostic boundaries Hum Mol Genet 2011 20 387 91 10.1093/hmg/ddq471 21037240 PMC3005906

[B6] Baum AE Akula N Cabanero M Cardona I Corona W Klemens B Schulze TG Cichon S Rietschel M Nöthen MM Georgi A Schumacher J Schwarz M Abou Jamra R Höfels S Propping P Satagopan J Detera-Wadleigh SD Hardy J McMahon FJ A genome-wide association study implicates diacylglicerol kinase (DGKH) and several other genes in the etiology of bipolar disorder Mol Psychiatry 2008 13 197 207 10.1038/sj.mp.4002012 17486107 PMC2527618

[B7] Ferreira MA O'Donovan MC Meng YA Jones IR Ruderfer DM Jones L Fan J Kirov G Perlis RH Green EK Smoller JW Grozeva D Stone J Nikolov I Chambert K Hamshere ML Nimgaonkar V Moskvina V Thase ME Caesar S Sachs GS Franklin J Gordon-Smith K Ardlie KG Gabriel SB Fraser C Blumenstiel B Defelice M Breen G Gill M Morris DW Elkin A Muir WJ McGhee KA Williamson R MacIntyre DJ McLean A St Clair D VanBeck M Pereira A Kandaswamy R McQuillin A Collier DA Bass NJ Young AH Lawrence J Ferrier IN Anjorin A Farmer A Curtis D Scolnick EM McGuYn P Daly MJ Corvin AP Holmans PA Blackwood DH Wellcome Trust Case Control Consortium (WTCCC) Gurling HM Owen MJ Purcell SM Sklar P Craddock NJ Collaborative genome-wide association analysis supports a role for ANK3 and CACNA1C in bipolar disorder Nat Genet 2008 40 1056 1058 10.1038/ng.209 18711365 PMC2703780

[B8] Schulze TG Detera-Wadleigh SD Akula N Gupta A Kassem L Steele J Pearl J Strohmaier J Breuer R Schwarz M Propping P Nothen MM Cichon S Schumacher J Rietschel M McMahon FJ Two variants in Ankyrin 3 (ANK3) are independent genetic risk factors for bipolar disorder Mol Psychiatry 2009 14 487 491 10.1038/mp.2008.134 19088739 PMC2793269

[B9] Lee MT Chen CH Lee CS Chen CC Chong MY Ouyang WC Chiu NY Chuo LJ Chen CY Tan HK Lane HY Chang TJ Lin CH Jou SH Hou YM Feng J Lai TJ Tung CL Chen TJ Chang CJ Lung FW Chen CK Shiah IS Liu CY Teng PR Chen KH Shen LJ Cheng CS Chang TP Li CF Chou CH Chen CY Wang KH Fann CS Wu JY Chen YT Cheng AT Genome-wide association study of bipolar I disorder in the Han Chinese population Mol Psychiatry 2011 16 548 56 10.1038/mp.2010.43 20386566

[B10] Athanasiu L Mattingsdal M Kähler AK Brown A Gustafsson O Agartz I Giegling I Muglia P Cichon S Rietschel M Pietiläinen OP Peltonen L Bramon E Collier D Clair DS Sigurdsson E Petursson H Rujescu D Melle I Steen VM Djurovic S Andreassen OA Gene variants associated with schizophrenia in a Norwegian genome-wide study are replicated in a large European cohort J Psychiatr Res 2010 44 748 53 10.1016/j.jpsychires.2010.02.002 20185149 PMC3224994

[B11] Bennett V Lambert S Physiological roles of axonal ankyrins in survival of premyelinated axons and localization of voltage-gated sodium channels Journal of Neurocytology 1999 28 303 318 10.1023/A:1007005528505 10739573

[B12] Kretschmer T England JD Happel LT Liu ZP Thouron CL Nguyen DH Beuerman RW Kline DG Ankyrin G and voltage gated sodium channels colocalize in human neurona - key proteins of membrane remodeling after axonal injury Neuroscience Letters 1999 323 151 155 10.1016/s0304-3940(02)00021-6 11950515

[B13] Dzhashiashvili Y Zhang Y Galinska J Lam I Grumet M Salzer JL Nodes of Ranvier and axon initial segments are ankyrin G-dependent domains that assemble by distinct mechanisms J Cell Biol 2007 177 857 70 10.1083/jcb.200612012 17548513 PMC2064285

[B14] Kizhatil K Davis JQ Davis L Hoffman J Hogan BL Bennett V Ankyrin-G is a molecular partner of E-cadherin in epithelial cells and early embryos J Biol Chem 2007 282 26552 61 10.1074/jbc.M703158200 17620337

[B15] Jenkins SM Bennett V Developing nodes of Ranvier are defined by ankyrin-G clustering and are independent of paranodal axoglial adhesion Proc Natl Acad Sci USA 2002 99 2303 8 10.1073/pnas.042601799 11842202 PMC122360

[B16] Jenkins SM Bennett V Ankyrin-G coordinates assembly of the spectrin-based membrane skeleton, voltage-gated sodium channels, and L1 CAMs at Purkinje neuron initial segments J Cell Biol 2001 155 739 46 10.1083/jcb.200109026 11724816 PMC2150881

[B17] Cruz DA Weaver CL Lovallo EM Melchitzky DS Lewis DA Selective alterations in postsynaptic markers of chandelier cell inputs to cortical pyramidal neurons in subjects with schizophrenia Neuropsychopharmacology 2009 34 12 24 10.1038/npp.2009.36 PMC2721024 19322171

[B18] Leonhard K Classification of endogenous psychoses and their differentiated etiology 1999 2 enlarged, Wien, New York Springer

[B19] Franzek E Beckmann H Different genetic background of schizophrenia spectrum psychoses: a twin study Am J Psychiatry 1998 155 76 83 9433342 10.1176/ajp.155.1.76

[B20] Becker T Knapp M Maximum-likelihood estimation of haplotype frequencies in nuclear families Genet Epidemiol 2004 27 21 32 10.1002/gepi.10323 15185400

[B21] Barrett JC Fry B Maller J Daly MJ Haploview: analysis and visualization of LD and haplotype maps Bioinformatics 2005 21 263 5 10.1093/bioinformatics/bth457 15297300

[B22] stastical power calculator http://www.dssresearch.com/toolkit/spcalc/power.asp

[B23] Pfuhlmann B Jabs B Althaus G Schmidtke A Bartsch A Stöber G Beckmann H Franzek E Cycloid psychoses are not part of a bipolar affective spectrum: results of a controlled family study J Affect Disord 2004 83 11 9 10.1016/j.jad.2004.03.005 15546641

[B24] Stöber G Franzek E Lesch KP Beckmann H Periodic catatonia: a schizophrenic subtype with major gene effect and anticipation Eur Arch Psychiatry Clin Neurosci 1995 245 135 41 10.1007/BF02193085 7669819

[B25] Liu Y Blackwood DH Caesar S de Geus EJ Farmer A Ferreira MA Ferrier IN Fraser C Gordon-Smith K Green EK Grozeva D Gurling HM Hamshere ML Heutink P Holmans PA Hoogendijk WJ Hottenga JJ Jones L Jones IR Kirov G Lin D McGuffin P Moskvina V Nolen WA Perlis RH Posthuma D Scolnick EM Smit AB Smit JH Smoller JW St Clair D van Dyck R Verhage M Willemsen G Young AH Zandbelt T Boomsma DI Craddock N O'Donovan MC Owen MJ Penninx BW Purcell S Sklar P Sullivan PF Meta-analysis of genome-wide association data of bipolar disorder and major depressive disorder Mol Psychiatry 2011 16 2 4 10.1038/mp.2009.107 20351715 PMC3883627

[B26] Quinn EM Hill M Anney R Gill M Corvin AP Morris DW Evidence for cis-acting regulation of ANK3 and CACNA1C gene expression Bipolar Disord 2010 12 440 5 10.1111/j.1399-5618.2010.00817.x 20636642

